# Supplementary figures and images for: Image-based flow simulation of platelet aggregates under different shear rates
Source: PLoS Comput Biol. 2023 Jul 10;19(7):e1010965. doi: 10.1371/journal.pcbi.1010965 (PMC10358939; doi:10.1371/journal.pcbi.1010965)

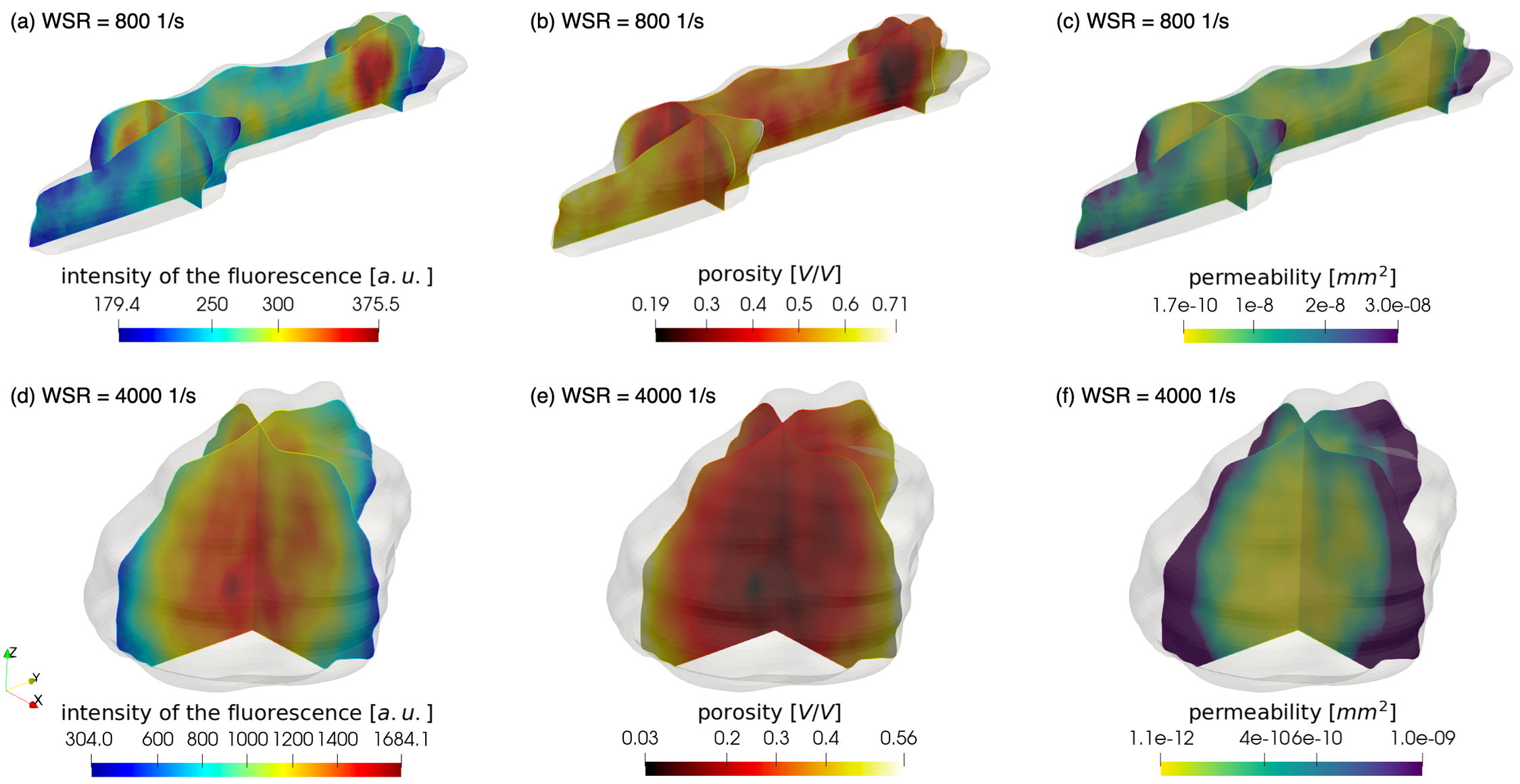

Supplement: S1 Fig — (a)-(c) Intensity of the fluorescence, corresponding porosity and permeability of the platelet aggregate on the cross-sections of the aggregate under 800 s−1 WSR. (d)-(f) Intensity of the fluorescence, corresponding porosity and permeability of the platelet aggregate on the cross-sections of the aggregate under 4000 s−1 WSR. (TIF) [file pcbi.1010965.s001.tif]

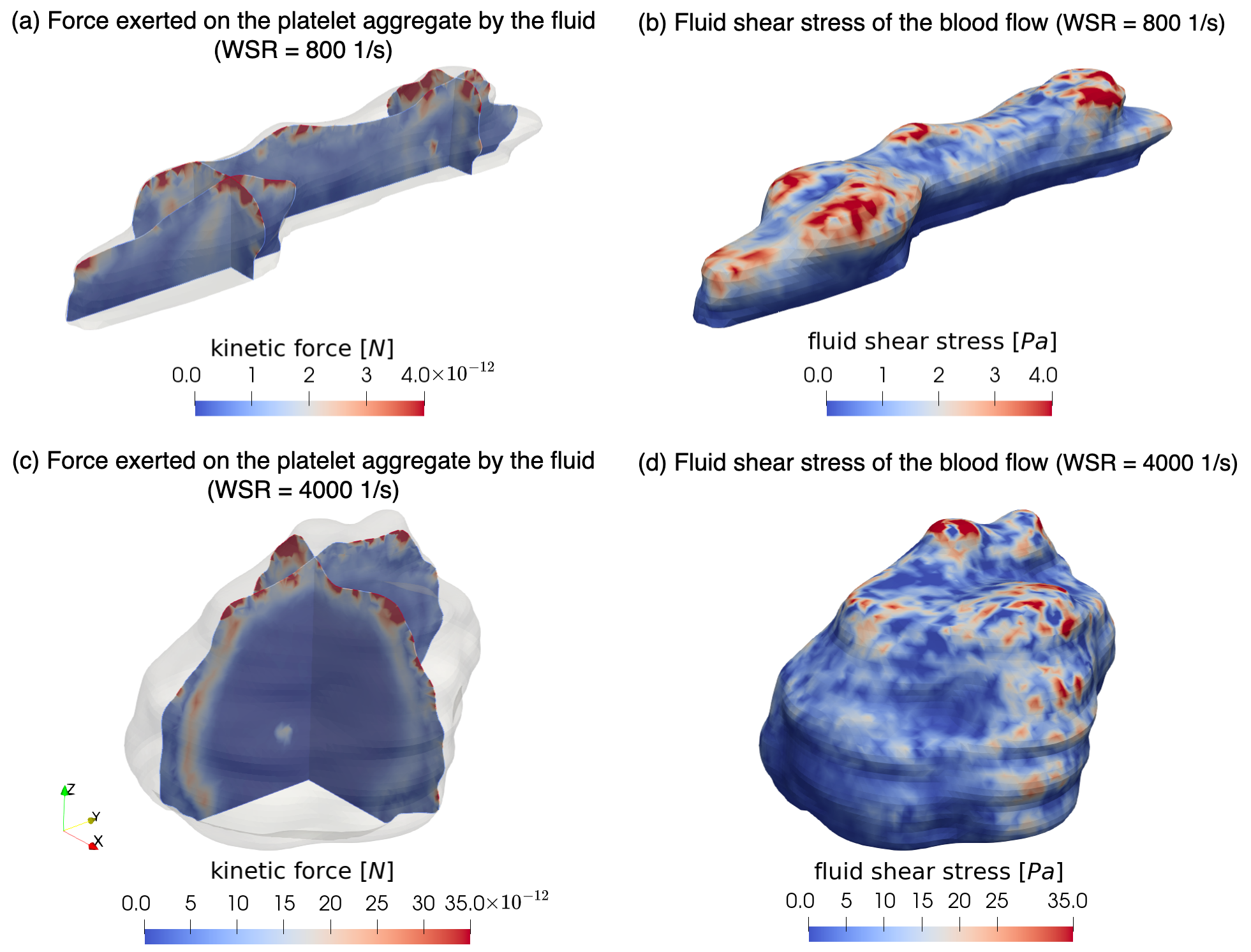

Supplement: S2 Fig — (a)-(b) The kinetic force exerted on the platelet aggregate and the fluid shear stress on the surface of the platelet aggregate under 800 s−1 WSR. (c)-(d) The kinetic force exerted on the platelet aggregate and the fluid shear stress on the surface of the platelet aggregate under 4000 s−1 WSR. (TIF) [file pcbi.1010965.s002.tif]

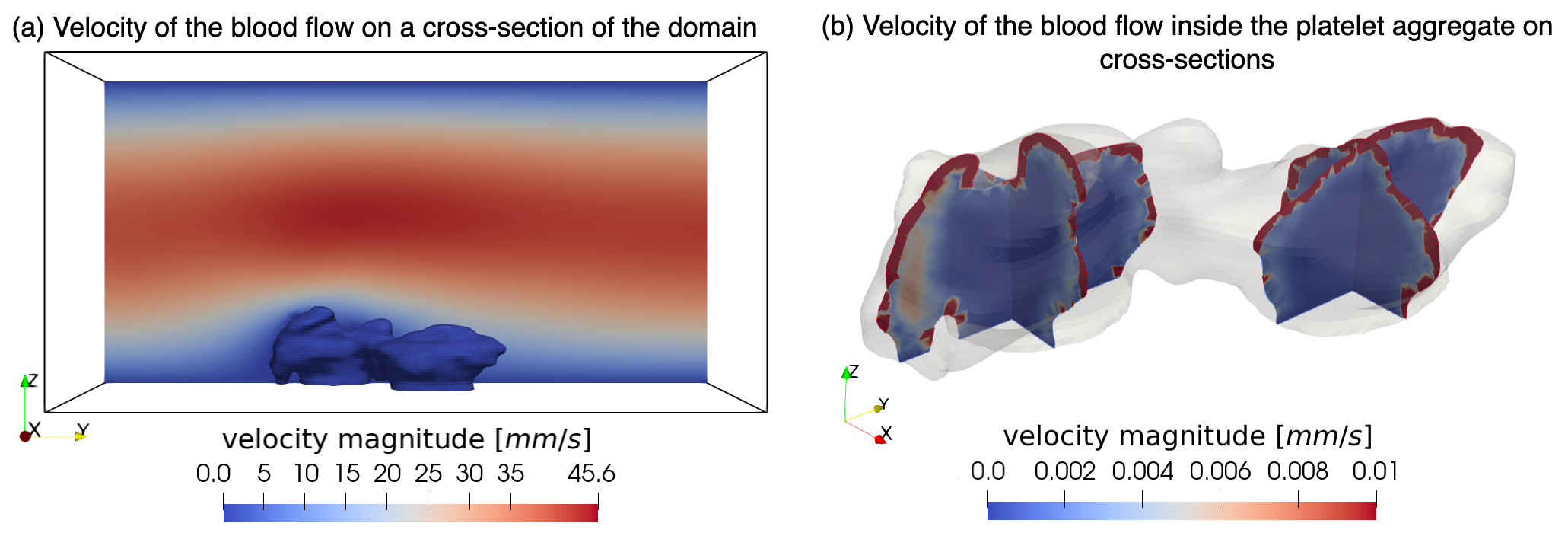

Supplement: S3 Fig — (a) The velocity field of the blood flow on a cross-section of the flow domain. (b) The velocity field of the blood flow inside the platelet aggregate on the cross-sections. (TIF) [file pcbi.1010965.s003.tif]
